# Outer membrane vesicles of the oral pathogen *Porphyromonas gingivalis* promote aggregation and phagocytosis of *Staphylococcus aureus*

**DOI:** 10.3389/froh.2022.948524

**Published:** 2022-07-22

**Authors:** Marines du Teil Espina, Anna Haider Rubio, Yanyan Fu, Marina López-Álvarez, Giorgio Gabarrini, Jan Maarten van Dijl

**Affiliations:** ^1^Department of Medical Microbiology, University of Groningen, University Medical Center Groningen, Groningen, Netherlands; ^2^Department of Dental Medicine, Karolinska Institutet, Huddinge, Sweden

**Keywords:** *Porphyromonas gingivalis*, PPAD, gingipains, outer membrane vesicles, *Staphylococcus aureus*, rheumatoid arthritis, *Staphylococcus aureus* bacteremia

## Abstract

*Staphylococcus aureus* is an opportunistic Gram-positive bacterial pathogen that causes a wide variety of infectious diseases, including *S. aureus* bacteremia (SAB). Recent studies showed that rheumatoid arthritis (RA) is a risk factor for SAB, as RA patients appear to be more susceptible to SAB and display higher degrees of disease severity or complications, such as osteoarticular infections. On the other hand, *Porphyromonas gingivalis* is a Gram-negative bacterial oral pathogen, which is notable for its implication in the etiopathogenesis of RA due to its unique citrullinating enzyme PPAD and its highly effective proteases, known as gingipains. Both PPAD and gingipains are abundant in *P. gingivalis* outer membrane vesicles (OMVs), which are secreted nanostructures that originate from the outer membrane. Here we show that *P. gingivalis* OMVs cause the aggregation of *S. aureus* bacteria in a gingipain- and PPAD-dependent fashion, and that this aggregation phenotype is reversible. Importantly, we also show that the exposure of *S. aureus* to OMVs of *P. gingivalis* promotes the staphylococcal internalization by human neutrophils with no detectable neutrophil killing. Altogether, our observations suggest that *P. gingivalis* can eliminate its potential competitor *S. aureus* by promoting staphylococcal aggregation and the subsequent internalization by neutrophils. We hypothesize that this phenomenon may have repercussions for the host, since immune cells with internalized bacteria may facilitate bacterial translocation to the blood stream, which could potentially contribute to the association between RA and SAB.

## Introduction

*Staphylococcus aureus* is a facultative anaerobic opportunistic Gram-positive bacterial pathogen encountered in humans and animals. Its predominant niches are the nasopharyngeal cavity, the skin and the gut, with nasal carriage in humans amounting up to 30% [1]. Altogether, it has been estimated that, through contamination, carriage, and colonization, this bacterium may contact up to 60% of the global population, and it should therefore be considered as part of the human microbiota [2–4]. Even though *S. aureus* carriage is usually asymptomatic, the vast array of niches that this bacterium can reach in the human body translates into an equally diverse spectrum of diseases, ranging from minor infections of the skin to biofilm-associated implant infections, septic arthritis affecting the joints, bacteremia, or endocarditis [5]. Additionally, *S. aureus* is one of the most common and lethal causative agents of nosocomial infections, as immunocompromised individuals are significantly more likely to succumb to this opportunistic pathogen. *S. aureus* is also a major contributor to the global burden of antimicrobial resistance (AMR), as in high-income countries about 25% of the AMR burden is linked to multi-drug resistant *S. aureus* [6]. One of the most lethal *S. aureus* infections is arguably *S. aureus* bacteremia (SAB), with an overall incidence of 20 to 50 cases/100,000 people depending on the population and a 30-day mortality rate of 30%, also due to the frequent occurrence of secondary infections [7, 8]. Interestingly, one of the risk factors for SAB is the autoimmune disease rheumatoid arthritis (RA) [9, 10]. The etiopathogenesis of this disease is still not fully understood, but it has been proposed that the periodontal keystone pathogen *Porphyromonas gingivalis* plays a major role in the onset of RA [11]. The unique citrullinating enzyme of this bacterium, the peptidylarginine deiminase PPAD, is in fact capable of citrullinating certain host proteins, which may trigger an autoimmune response in genetically predisposed individuals [11]. This may then result in the formation of autoantibodies against the citrullinated proteins, termed ACPAs, that are highly specific for RA [12, 13]. Importantly, this citrullination is largely dependent on the activity of arginine- and lysine-specific proteases of *P. gingivalis*, known as the gingipains RgpA/B and Kgp. In particular, the cleavage of a protein by RgpA/B will expose a carboxyl-terminal arginine residue that is preferentially targeted by PPAD. Much research has therefore been focused on these gingipains, PPAD, and the mechanisms by which these enzymes are secreted by *P. gingivalis* [11, 14, 15]. One of the most notable secretion pathways for PPAD and gingipains involves the sorting into outer membrane vesicles (OMVs), which are outer membrane blebbings that carry a cargo of virulence factors, allowing easy delivery to targeted host cells [16]. Interestingly, the released OMVs may also interact with the human microbiome. Thus, OMVs of *P. gingivalis* have been implicated in the aggregation of *S. aureus* cells, which is potentially facilitated by the coexistence of these two bacterial species in certain niches within the oral cavity and nasopharynx [17–21]. This, combined with the notion that RA is a risk factor for SAB and that *S. aureus* is often found in patients with periodontitis [22–24], prompted us to further characterize this aggregation phenomenon.

## Materials and methods

### Bacterial cultures

Culturing of the *P. gingivalis* strain W83 and the respective PPAD deletion mutant [25] was performed as described by Stobernack et al. [24]. The bacteria were streaked on Blood Agar No. 2 (BA2) plates and grown under anaerobic conditions (10% CO_2_, 10% H_2_, 80% N_2_) for 5 days at 37°C. The bacteria were subsequently used to inoculate enriched Brain-Heart Infusion (BHI) broth containing 0.5 mg/mL L-cysteine, 0.5 mg/mL hemin and 10 μg/mL menadione. The cultures were incubated anaerobically at 37°C for 20 h before use. Optical density was measured at a wavelength of 600 nm (OD_600_) as the stationary growth phase was reached. *S. aureus* strain HG001 carrying plasmid pJL-sar-GFP to express the green fluorescent protein [GFP; [26]] was cultured in Tryptic Soy Broth (TSB) with erythromycin (10 μg/mL; Sigma Aldrich, Burlington, Massachusetts, USA). Upon overnight incubation at 37°C with shaking at 220 rpm, the OD_600_ was measured and the bacteria were used to inoculate Roswell Park Memorial Institute 1,640 (RPMI) medium supplemented with 2 mM L-glutamine at an OD_600_ of 0.05. Subsequently, the culture was incubated in a shaking water bath at 37°C and 220 rpm until the OD_600_ reached 0.5. The clinical *Aggregatibacter actinomycetemcomitans* isolate 30R [27], used as positive control for the cytotoxicity assays, was grown on BHI agar plates supplemented with 5% L-cysteine, 5 mg/L hemin and 1 mg/L menadione, at 37°C and in 5% CO_2_. Several colonies of *A. actinomycetemcomitans* were picked from a BHI plate to inoculate BHI broth and the bacteria were grown overnight in standing culture to mid-exponential phase (OD_600_ of ~0.5).

### *P. gingivalis* OMV isolation, purification and quantification

*P. gingivalis* bacterial cells were removed from cultures in the early stationary phase by centrifugation at 8,000 x *g* for 15 min at 4°C. Subsequently, the resulting supernatant was filtered using a 0.22 μm filter (GE Healthcare Life Sciences, Chicago, Illinois, USA) and ultra-centrifuged overnight at 100,000 x *g* and 4°C using an Optima^TM^ XL-80K ultracentrifuge (Beckman Coulter Inc., Brea, California, USA). The OMV pellet was then resuspended in 4 mL phosphate-buffered saline (PBS) and ultra-centrifuged again at 100,000 x *g* for 2 h at 4°C. The resulting OMV pellet was resuspended in 400 μL of a 50% ‘OptiPrep^TM^ Density Gradient Medium' iodixanol solution (Sigma-Aldrich, Burlington, Massachusetts, USA) diluted with Buffer A composed of 60 mM HEPES-NaOH and 0.85 % (w/v) NaCl at pH 7.4. For subsequent OptiPrep layers (40%, 30%, 20% and 10%) “buffer B” was used as diluent, which was composed of 0.85% (w/v) NaCl and 10 mM HEPES-NaOH. The different Optiprep layers of 900 μL each were carefully stacked on top of the OMV-containing layer, with the upper layer having the lowest concentration of Optiprep (10%). The samples were then centrifuged for 2 h at 100,000 x *g* and 4°C. After centrifugation, the middle fractions, corresponding to the 20 and 30% Optiprep solutions, were pooled together as they contained the purified OMVs. To identify the OMV-containing Optiprep fractions, a trichloroacetic acid (TCA) precipitation of all collected fractions was performed and the precipitated proteins were analyzed by Western Blotting using an antibody specific for Omp41 (GP2451)[28]. Subsequently, to remove the Optiprep, the pooled OMV-containing fractions were diluted in 4 mL of buffer B and ultra-centrifuged at 100,000 x *g* and 4°C overnight. The next day, the supernatant was discarded, the OMV pellet was resuspended in 4 mL PBS and the centrifugation step was repeated for 2 h. Lastly, the OMVs were resuspended in PBS, and sterilized using a 0.22 μm filter. The OMV aliquots were then stored at −80°C until further use. For quantification, the Pierce^TM^ bicinchoninic acid (BCA) Protein Assay Kit (Thermo Fisher, Waltham, Massachusetts, USA) was used to determine the OMV protein concentration. To solubilize the OMVs for protein quantification, 2% SDS was used as recommended previously [29].

### Human neutrophils

Fresh blood donations were used to obtain human primary neutrophils, which were obtained from female donors aged 29 to 31 that had been medically examined beforehand. The blood was collected in EDTA-coated tubes and diluted 1:1 with PBS. Subsequently, the blood was layered on top of Lymphoprep^TM^ buffer (StemCell Technologies, Vancouver, Canada) in a ratio of 2:1 blood and lymphoprep, respectively. After centrifugation at 2,500 rpm for 20 min at room temperature (RT) without a break, the top layer containing the plasma was carefully transferred to a separate tube and kept on ice. Remaining lymphoprep and the monocyte cell layer were discarded. Erythrocytes present in the bottom layer with neutrophils were removed by lysis with 1 × RBC lysis buffer (BioLegend, San Diego, California, USA). After being kept on ice and shaking for 10 min, the tubes were centrifuged at 400 × *g* for 5 min. The supernatant containing the lysed erythrocytes was removed before performing a second lysis step. The purified neutrophils were then resuspended in RPMI (Gibco, Waltham, Massachusetts, USA) supplemented with 2 mM L-glutamine and 10% autologous donor plasma. Cell viability was assessed by Trypan Blue staining.

### Neutrophil infection with *S. aureus* HG001 and OMVs of *P. gingivalis*

A total of 5 ×10^5^ neutrophils in supplemented RPMI (2mM L-glutamine and 10% autologous serum) were seeded in each well of a 24-well cell culture plate, with or without sterile coverslips for subsequent analysis by either immunostaining or flow cytometry. To study the effect of *P. gingivalis* OMVs on the interaction of *S. aureus* with neutrophils, 5 μg of OMVs of *P. gingivalis* were pre-mixed and incubated with *S. aureus* for 15 min at 37°C and, subsequently, added to each well of the 24-well cell culture plate with the neutrophils. A standardized amount of 5 μg of OMVs per experiment was used following optimization experiments with 1 to 10 μg of OMVs per experiment. A multiplicity of infection (MOI) of 15 was used for all samples. The neutrophil infection was allowed to proceed for 60 min at 37°C and 5% CO_2_. Non-internalized *S. aureus* bacteria were eliminated by the addition of lysostaphin (25 μg/mL; AMBI Products, Rockville, Maryland, USA) and incubation for another 60 min under the same conditions [30]. After incubation, cells were either fixed on the coverslips before immunostaining or prepared for flow cytometry. Cell fixation for fluorescence microscopy was performed by incubation with 4% paraformaldehyde (PFA) for 15 min. Subsequently, the coverslips were washed twice with PBS. For the flow cytometry experiments, cells were collected from each well by repeated up-and-down pipetting. Subsequently, the cells were transferred to a 1.5 mL Eppendorf tube. PFA 4% was then added and fixation was allowed to proceed for 15 min at RT. The PFA was then removed by washing the cells once with PBS. Cells were re-suspended in 300 μL of PBS and stored at 4°C until analysis by flow cytometry on the following day.

### Antibodies against whole-cell *P. gingivalis*

Polyclonal antibodies against whole *P. gingivalis* W83 cells were raised at Eurogentec (Seraing, Belgium). To this end, the bacteria were fixed with PFA 1% and washed five times with PBS prior immunization of a rabbit following a protocol provided by Eurogentec. Selection of the rabbit to be immunized was performed based on the screening of pre-immune sera without cross-reactivity toward *P. gingivalis*. Subsequently, the specificity of the obtained polyclonal antibodies was verified by Western blotting and immunostaining.

### Immunostaining

Coverslips with fixed neutrophils and bacteria were first washed once with PBS. Following this step, the neutrophil membranes were permeabilized using 0.5% Tween-20 for 15 min at RT. Subsequently, a blocking step was performed with 1% bovine serum albumin (BSA) for 1 h at RT. Incubation with primary rabbit polyclonal antibodies raised against whole *P. gingivalis* cells was performed for 1 h at RT, which was followed by two washes with PBS. Subsequently, incubation with either AlexaFluor488, AlexaFluor647 or AlexaFluor555 goat anti-rabbit antibodies (Invitrogen, Waltham, Massachusetts, USA) was performed for 30 min at RT in the dark. Neutrophil nuclei were stained at this step with 4′,6-diamidino-2-phenylindole (DAPI; Sigma-Aldrich). In some experiments, actin was visualized using tetramethyl-rhodamine B isothiocyanate-phalloidin (TRITC-phalloidin; Sigma-Aldrich). After washing 2 times with PBS, the coverslips were mounted on Polylysine Slides (Thermo Fisher) using Mowiol 4-80 as the mounting medium (Sigma-Aldrich). All washing steps and incubations were done carefully to avoid detachment of cells, especially neutrophils, from the coverslips. The mounting medium was dried by overnight incubation at RT in the dark.

### Confocal fluorescence microscopy

Images were recorded with a Leica SP8 confocal microscope (Leica Microsystems, Wetzlar, Germany) and analyzed using the LAS X software. For selected images, three-dimensional reconstructions from Z-stacks of two-dimensional confocal microscopy images were made using the Imaris 7.6.5 software (Oxford Instruments, Abingdon, UK).

### Flow cytometry

Flow cytometric quantification of *S. aureus*-infected neutrophils was performed with a Cytoflex S flow cytometer (Beckman Coulter) by excitation of GFP with a 488 nm laser and detection at 525/40 nm. Analysis of the flow cytometry data was performed with Kaluza Analysis Software (Beckman Coulter) using the gating strategy previously described [30]. The percentage of GFP-positive cells and the mean fluorescence intensity were used to calculate the adherence and internalization indices as previously described [31]. Results are presented as the ratios of the adherence or internalization indices in the presence vs. the absence of OMVs.

### LDH cytotoxicity assay

Neutrophil cytotoxicity after *S. aureus* infection (+/– OMVs of *P. gingivalis*) was measured by determining the extracellular lactate dehydrogenase (LDH) activity using the CyQUANT™ LDH Cytotoxicity Assay Kit (Thermo Fisher) following the manufacturer's instructions. 3.5 x 10^4^ neutrophils in 100 μL of supplemented RPMI medium (2 mM L- glutamine, 10% autologous serum) were seeded in wells of a 96-well tissue culture plate and the plate was subsequently incubated for 30 min at 37°C and 5% CO_2_. To obtain the maximum LDH activity control, 10 μL of 10X lysis buffer was added to the designated wells and incubated for 45 min at 37°C and 5% CO_2_. To obtain the spontaneous LDH activity control, 10 μL of sterile ultrapure Milli-Q (MQ) water was added per well. Simultaneously, *S. aureus* cells were pre-incubated with or without OMVs of *P. gingivalis* (from strains W83 and W83ΔPPAD) in RPMI medium (2 mM L-glutamine, 10 % autologous serum) for 15 min at 37°C. For the *S. aureus* infection experiments, an MOI of 20 was used, which corresponds to 5.3 x 10^5^ colony-forming units (CFUs) and 0.35 μg of OMVs. Subsequently, 10 μL of the *S. aureus* cells with or without *P. gingivalis* OMVs were added to the designated wells. As a positive control for bacterial cytotoxicity, the highly leukotoxic clinical *A. actinomycetemcomitans* isolate 30R was used at a MOI of 100 [27]. The plate was incubated for 60 min at 37°C and 5% CO_2_. Next, 50 μL of each sample was transferred to another 96-well plate and, subsequently, 50 μL of the reaction mixture was added to each well and the plate was incubated at RT for 30 min, protected from light. Lastly, 50 μL of stop solution was added to each well and mixed by gentle tapping. The absorbance was measured at 490 and 680 nm to calculate the cytotoxicity percentage. For each sample, two biological replicates and three technical replicates were used.

To determine LDH activity, the 680 nm absorbance value (background) was subtracted from the 490 nm absorbance value before calculation of the % cytotoxicity by the following formula:


(1) $$\begin{array}{l} \text{\%Cytotoxicity=[(treatedLDHactivity}\\ -\text{SpontaneousLDHActivity)/(MaximumLDHactivity}\\ -\text{SpontaneousLDH}\;\text{activity})]\times 100 \end{array}$$

### *S. aureus* growth measurements

*S. aureus* was cultured in RPMI medium until an OD_600_ of 0.5 was reached. The bacterial culture was then diluted to an OD_600_ of 0.05, and 100 μl aliquots of this suspension were transferred to a 96-well plate. Bacterial growth was measured by OD_600_ readings every 15 min with continuous shaking at 37°C in a Synergy two multi-mode microplate reader (BioTek Instruments, Inc., Winooski, VT). Upon reaching the early or late exponential growth phase, 5 μg of OMVs from *P. gingivalis* W83 or W83ΔPPAD were added. The respective growth curves were visualized using GraphPad Prism version 8.0 for Windows (GraphPad Software, San Diego, California, USA). The effect of OMVs on exponentially growing bacteria was assessed by recording the growth pattern of OMV-treated bacterial cells in comparison with untreated bacteria. Three replicates were used for each condition and an average of the individual values was used to visualize each growth curve. To assess the effect of gingipains on staphylococcal aggregation, 100 μM of gingipain inhibitors were added simultaneously with the OMVs. The gingipain inhibitors used were the Kgp inhibitor Cathepsin B inhibitor II (Merck, Kenilworth, New Jersey, USA) and the RgP inhibitor Leupeptin (Sigma-Aldrich). To verify the possible adaptation of *S. aureus* to aggregation mediated by *P. gingivalis* OMVs, the bacterial cells that had been exposed to OMVs were collected after reaching stationary phase and used for another round of culturing in the absence or presence of OMVs of *P. gingivalis*.

To visualize the aggregation of *S. aureus*, an Amersham Typhoon NIR imaging system was used for scanning the wells with or without aggregated GFP-expressing bacteria. In addition, to assess the localization of the OMVs within the *S. aureus* aggregates, the aggregated bacteria were transferred to another 96-well plate. The OMVs were then immunostained and visualized by confocal fluorescence microscopy as described above.

### Medical ethical approval

Blood donations from healthy volunteers were collected with approval of the medical ethical committee of the University Medical Center Groningen (approval no. Metc2012-375) after written informed consent and in accordance with the Helsinki Guidelines.

### Statistical analyses

Data were analyzed using GraphPad Prism Version 8.0 for Windows (GraphPad Software). Statistical significance was determined using a paired two-tailed Student's *T*-test. A *P*-value of ≤ 0.05 was considered as significant.

## Results

### Reduced CFU counts upon incubation of *S. aureus* with OMVs of *P. gingivalis*

**Figure 1:**
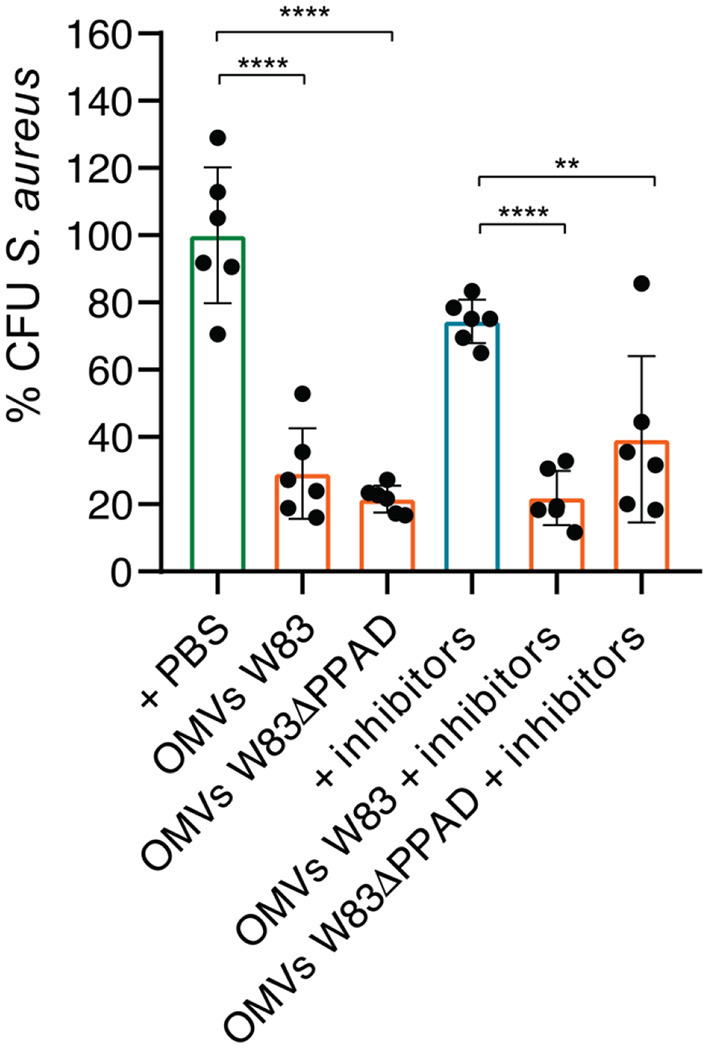
Exposure to *P. gingivalis* OMVs decreases *S. aureus* CFU counts. *S. aureus* HG001 was incubated for 90 min with OMVs of *P. gingivalis* W83 or W83ΔPPAD in the presence or absence of the gingipain inhibitors Cathepsin B inhibitor II (inhibiting KgP) and Leupeptin (inhibiting RgPA/B). All groups were compared to the control of *S. aureus* in PBS. The mean and standard deviations were calculated from three biological replicates and two technical replicates. Statistical significance of observed differences was evaluated with two-tailed unpaired Student's *t*-tests. **, *P* < 0.01; ****, *P* < 0.0001.

In view of the connection between SAB and rheumatoid arthritis [9, 10], and the role that *P. gingivalis* plays in the etiology of RA [11], we decided to investigate the effects of *P. gingivalis* OMVs on *S. aureus* cells with a focus on the virulence factors of *P. gingivalis* that have been implicated in RA pathogenesis, especially PPAD and the gingipains. To this end, *S. aureus* HG001 was incubated for 90 min in the presence or absence of OMVs from the *P. gingivalis* type strain W83 or the respective PPAD deletion mutant (W83ΔPPAD). We used RPMI medium for this and subsequent experiments, as we have previously shown that the global transcript profile of *S. aureus* HG001 grown in RPMI is very similar to the global transcript profile of this strain upon growth in human plasma [32]. Subsequently, the bacteria were plated on blood agar. This revealed a significant decrease in the CFU counts of *S. aureus* HG001 upon incubation with OMVs as compared to the control group that was mock-treated with PBS (Figure 1). To determine whether gingipain activity might contribute to the observed CFU reduction, the gingipain inhibitors leupeptin and cathepsin B inhibitor II that, respectively, block the activity of RgpA/B and Kgp were added [33]. However, the addition of these inhibitors did not prevent the reduction of *S. aureus* CFUs caused by the presence of *P. gingivalis* OMVs (Figure 1). Nevertheless, there was a trend toward a less severe CFU reduction upon incubation of *S. aureus* with OMVs of the W83ΔPPAD strain in the presence of gingipain inhibitors (Figure 1).

### *P. gingivalis* OMVs induce aggregation of *S. aureus* in a PPAD- and gingipain-dependent manner

**Figure 2:**
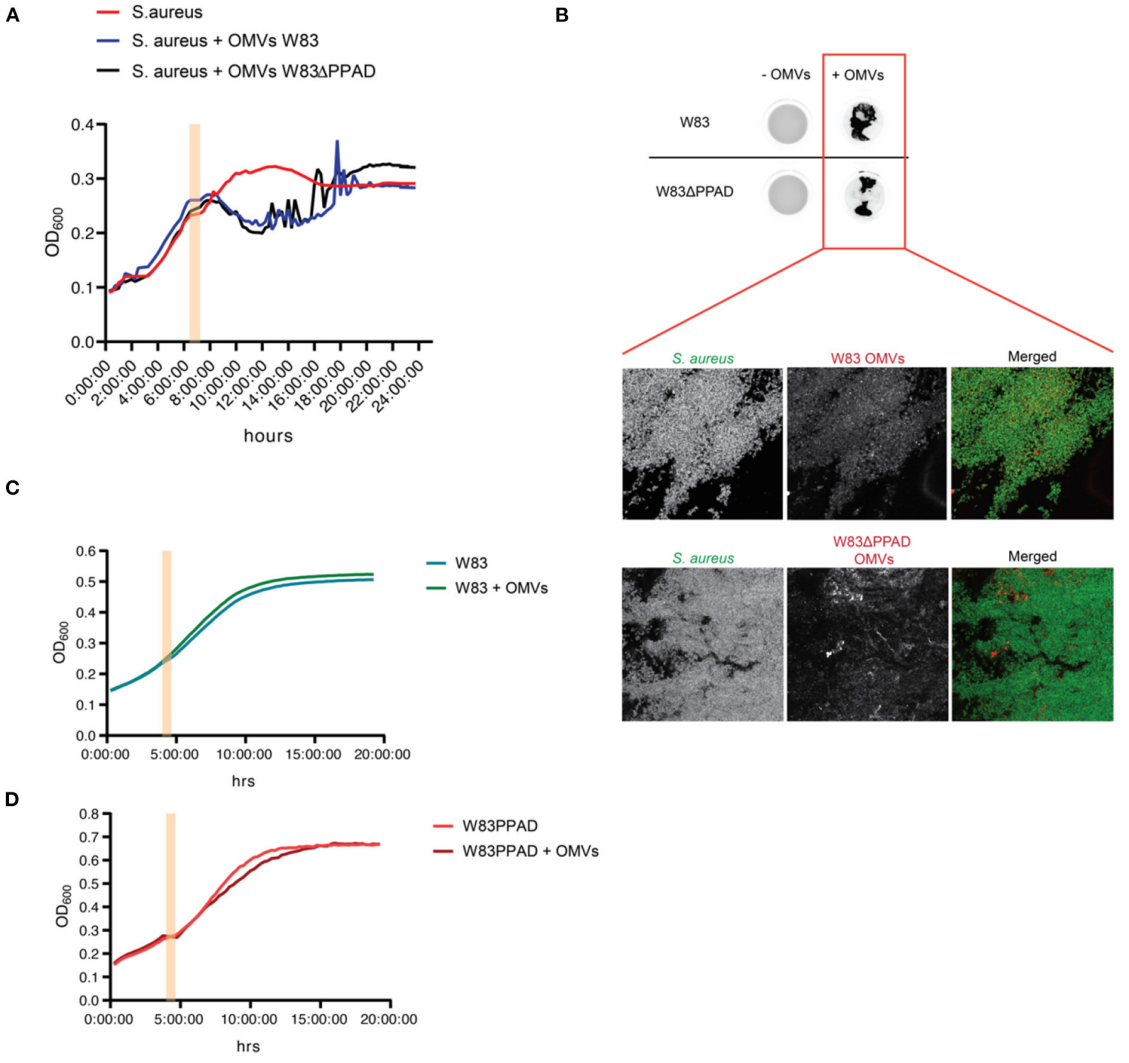
*S. aureus* aggregation upon exposure to OMVs of *P. gingivalis* W83 or W83ΔPPAD. **(A)** Growth curves of *S. aureus* HG001 in microtiter plates with or without added OMVs of *P. gingivalis* W83 or W83ΔPPAD at mid-exponential phase. Noise in the growth curves reflects bacterial aggregation. **(B)** Imaging of OMV-induced *S. aureus* aggregation in microtiter plate wells upon OMV exposure and verification of the bacterial aggregation by confocal fluorescence microscopy. The microscopy images show colocalization of the *S. aureus* aggregates with OMVs. *S. aureus* was visualized by the expression of GFP from plasmid pJL-sar-GFP and OMVs were labeled with specific antibodies (scale bars = 50 μm). **(C,D)** Growth of *P. gingivalis* W83 or W83ΔPPAD with or without added autologous OMVs. Pink bars in the growth curves mark the addition of OMVs to the cultures. Each condition involved four biological replicates and one technical replicate.

To verify whether the *S. aureus* CFU count decreased due to killing by the OMVs, or rather due to bacterial aggregation as previously reported by Kamaguchi et al. [17], growth experiments of *S. aureus* in the absence or presence of *P. gingivalis* OMVs were performed. In particular, *S. aureus* HG001 was challenged with OMVs of *P. gingivalis* W83 or the respective PPAD deletion mutant W83ΔPPAD during exponential growth. As shown in Figure 2A, the addition of OMVs caused a temporal drop in the OD_600_, but the bacteria were able to resume growth, ultimately reaching an OD_600_ that was comparable to that of the untreated bacteria. This indicated that the *P. gingivalis* OMVs did not kill *S. aureus*. In fact, drastic fluctuations in the OD_600_ measurements were observed over time when OMVs were added to the *S. aureus* cultures, which was indicative of the formation of *S. aureus* aggregates within the wells. To verify this, the wells were inspected for GFP fluorescence emitted by the bacteria due to the presence of plasmid pJL-sar-GFP using a fluorescence imaging system. This revealed the presence of aggregated bacteria in the wells supplemented with OMVs (Figure 2B). Moreover, the OMVs were localized within the *S. aureus* aggregates, as was shown by immunostainings of the aggregated bacteria collected from each well (Figure 2B). In contrast to *S. aureus*, the addition of autologous OMVs to *P. gingivalis* W83 or W83ΔPPAD did not detectably affect the growth of these two strains (Figures 2C,D).

**Figure 3:**
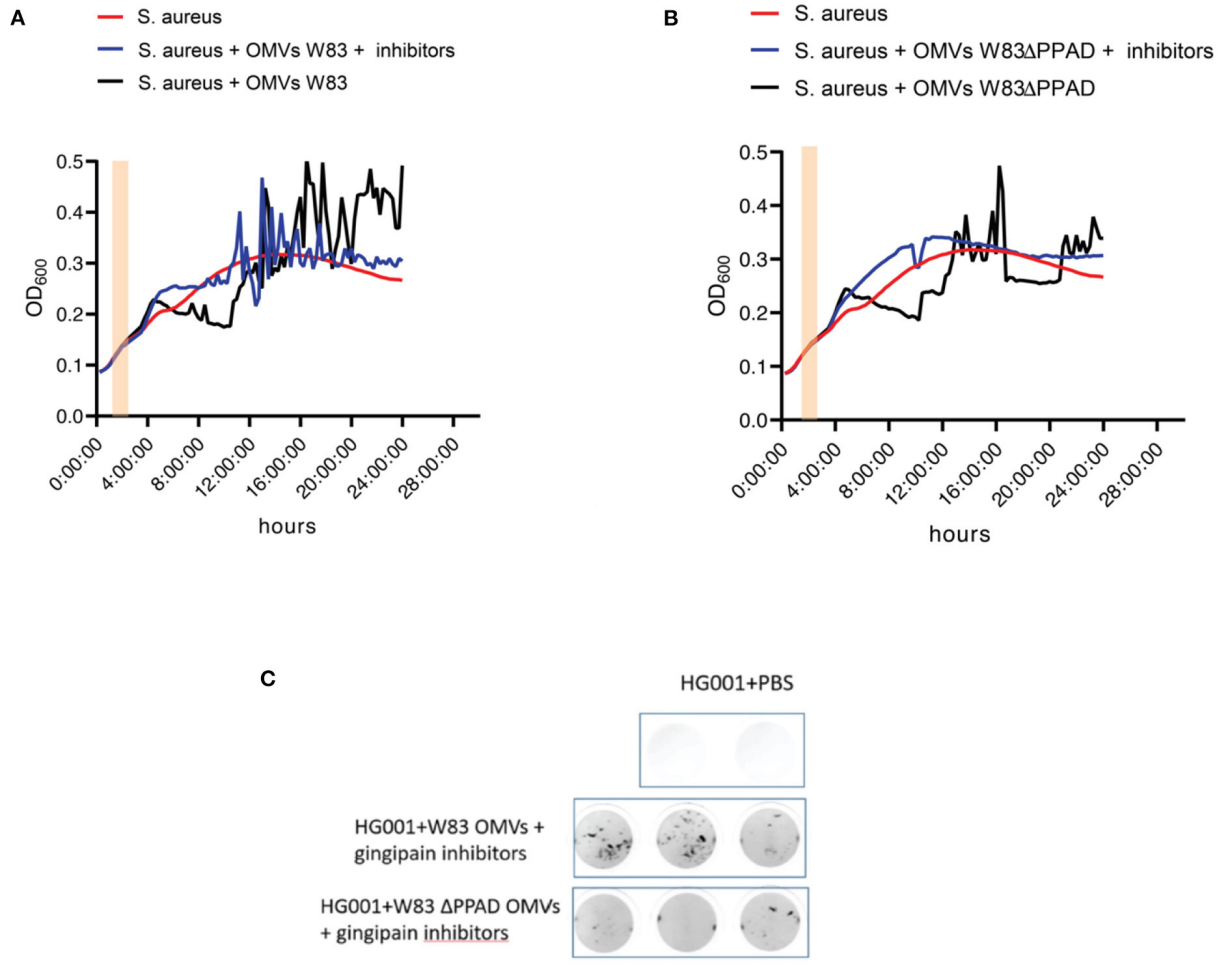
OMV-mediated *S. aureus* aggregation is suppressed by the absence of PPAD and gingipain activity. **(A)** Growth curves of *S. aureus* HG001 with or without added OMVs of *P. gingivalis* W83 grown in the presence or absence of the gingipain inhibitors Cathepsin B inhibitor II and Leupeptin. **(B)** Growth curves of *S. aureus* HG001 with or without added OMVs of *P. gingivalis* W83ΔPPAD grown in the presence or absence of the gingipain inhibitors as in **(A). (C)** Scans of *S. aureus* aggregates in microtiter plate wells upon OMV exposure in the presence of gingipain inhibitors. Pink bars in the growth curves mark the addition of OMVs to the cultures. Each condition involved four biological replicates and one technical replicate.

Interestingly, OMVs of the PPAD deletion mutant caused less severe aggregation than the wild-type OMVs (Figure 2B), suggesting a role for PPAD in the aggregation process. Knowing the importance of gingipains in the lifestyle of *P. gingivalis*, including the citrullination of bacterial and host proteins by PPAD, the effect of gingipain activity on OMV-induced *S. aureus* aggregation was also tested in growth experiments (Figure 3). As shown in Figure 3, addition of the gingipain-specific inhibitors leupeptin and cathepsin B inhibitor II did reduce the OMV-induced aggregation of *S. aureus* over time, which is reflected both in the less fluctuating OD_600_ readings (Figures 3A,B) and the scans of the respective microtiter plate wells (Figure 3C). Furthermore, when PPAD was absent from the OMVs, the aggregation of *S. aureus* was strongly suppressed by the addition of gingipain inhibitors (Figures 3B,C). Together, these observations show that both PPAD and gingipains contribute to the OMV-induced aggregation of the *S. aureus* bacteria. Considering the different aggregation levels caused by either the absence of PPAD or the addition of only the gingipain inhibitors, we conclude that PPAD and gingipains have synergistic roles in this phenomenon. This is fully consistent with the notion that gingipains are required for PPAD to citrullinate bacterial and host proteins [11, 14].

### The aggregation of *S. aureus* induced by *P. gingivalis* OMVs is fully reversible

**Figure 4:**
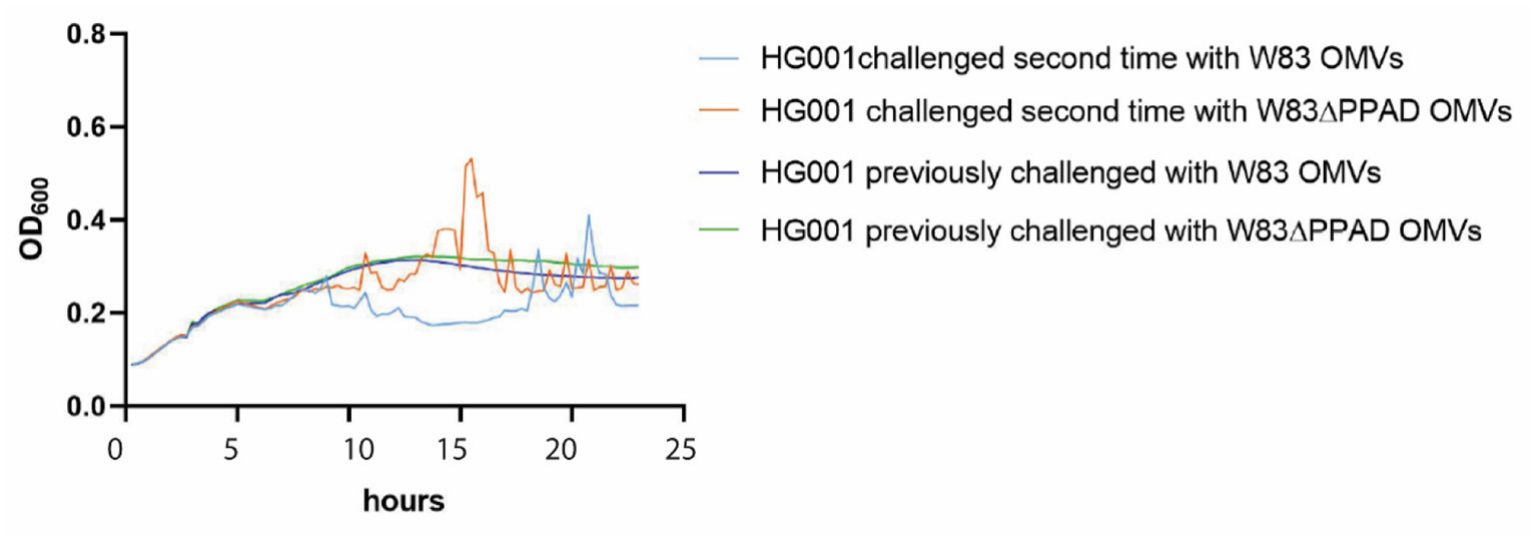
OMV-mediated aggregation of *S. aureus* is a reversible phenotype. *S. aureus* HG001 was exposed to OMVs of *P. gingivalis* W83 or W83ΔPPAD OMVs. The aggregated bacteria were collected and re-cultured in fresh RPMI medium with or without the addition of OMVs after 5 h of growth. The growth curves labeled as “previously challenged” refer to growth in the absence of OMVs. Each condition involved four biological replicates and one technical replicate.

To test whether the OMV-induced aggregation of *S. aureus* is a reversible phenotype, an experiment was performed in which *S. aureus* cells that had aggregated in the presence of *P. gingivalis* OMVs were isolated and subsequently cultured for a second time without or with OMVs of *P. gingivalis*. As shown in Figure 4, these *S. aureus* bacteria resumed normal growth in the absence of OMVs, whereas they displayed the aggregation phenotype when challenged for a second time with OMVs from *P. gingivalis* W83 or W83ΔPPAD. This shows that the aggregation phenotype displayed by *S. aureus* in the presence of *P. gingivalis* OMVs is fully reversible.

### *P. gingivalis* OMV-induced neutrophil adhesion and internalization of *S. aureus*

**Figure 5:**
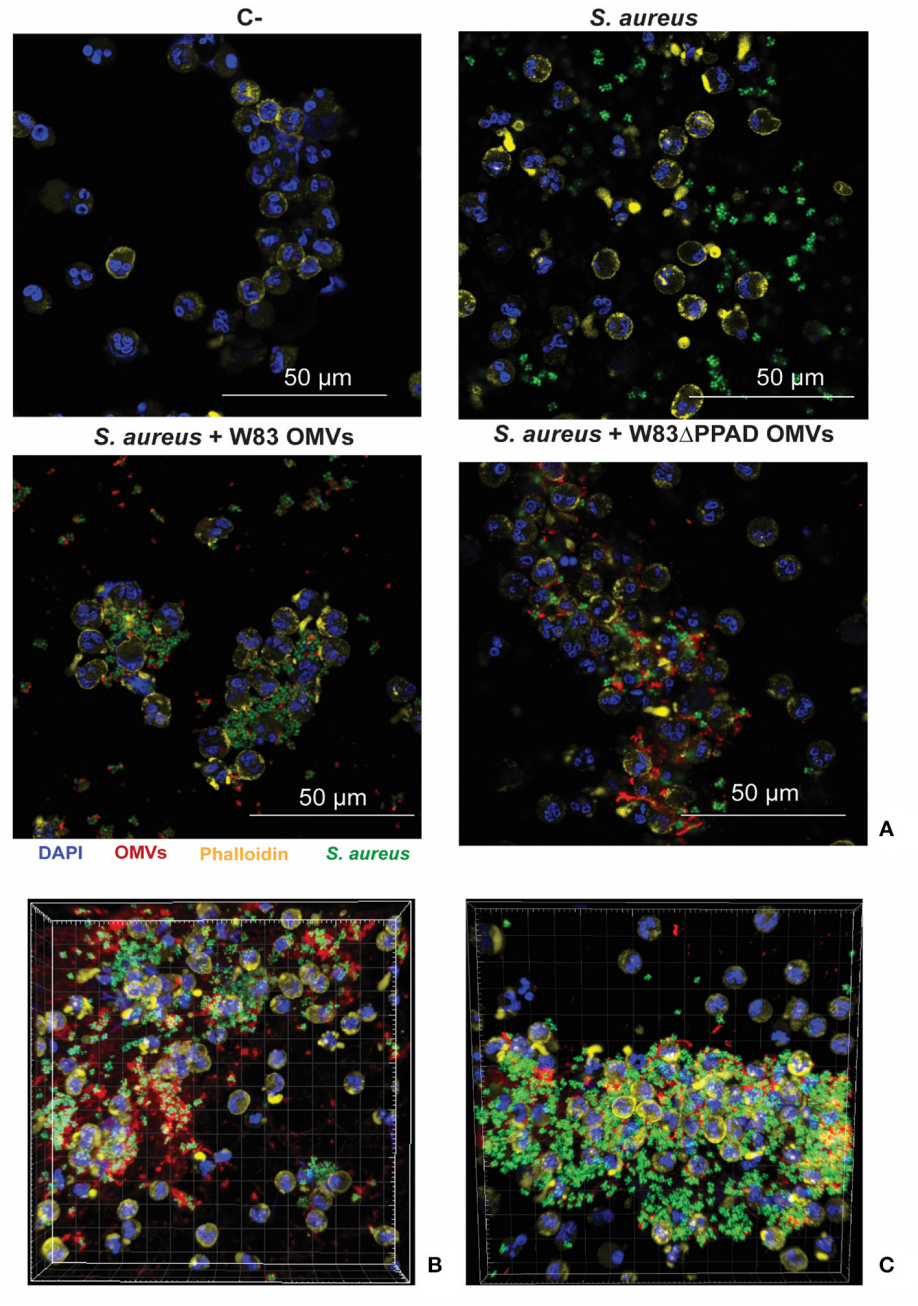
Neutrophil association and internalization of *S. aureus* upon exposure to *P. gingivalis* OMVs. **(A)** Representative confocal microscopy images of neutrophils infected with *S. aureus* HG001 that was exposed to *P. gingivalis* OMVs. **(B,C)** Three-dimensional reconstructions of confocal fluorescence microscopy images of neutrophils incubated with *S. aureus* in the presence of OMVs from *P. gingivalis* W83 **(B)** or OMVs from *P. gingivalis* W83ΔPPAD. **(C)** DAPI was used to stain the nuclei of the neutrophils and Phalloidin-TRITC to stain actin. *S. aureus* was detected based on GFP expression from plasmid pJL-sar-GFP and OMVs were labeled with specific antibodies (scale bars = 50 μm). Each experiment was repeated at least twice.

**Figure 6:**
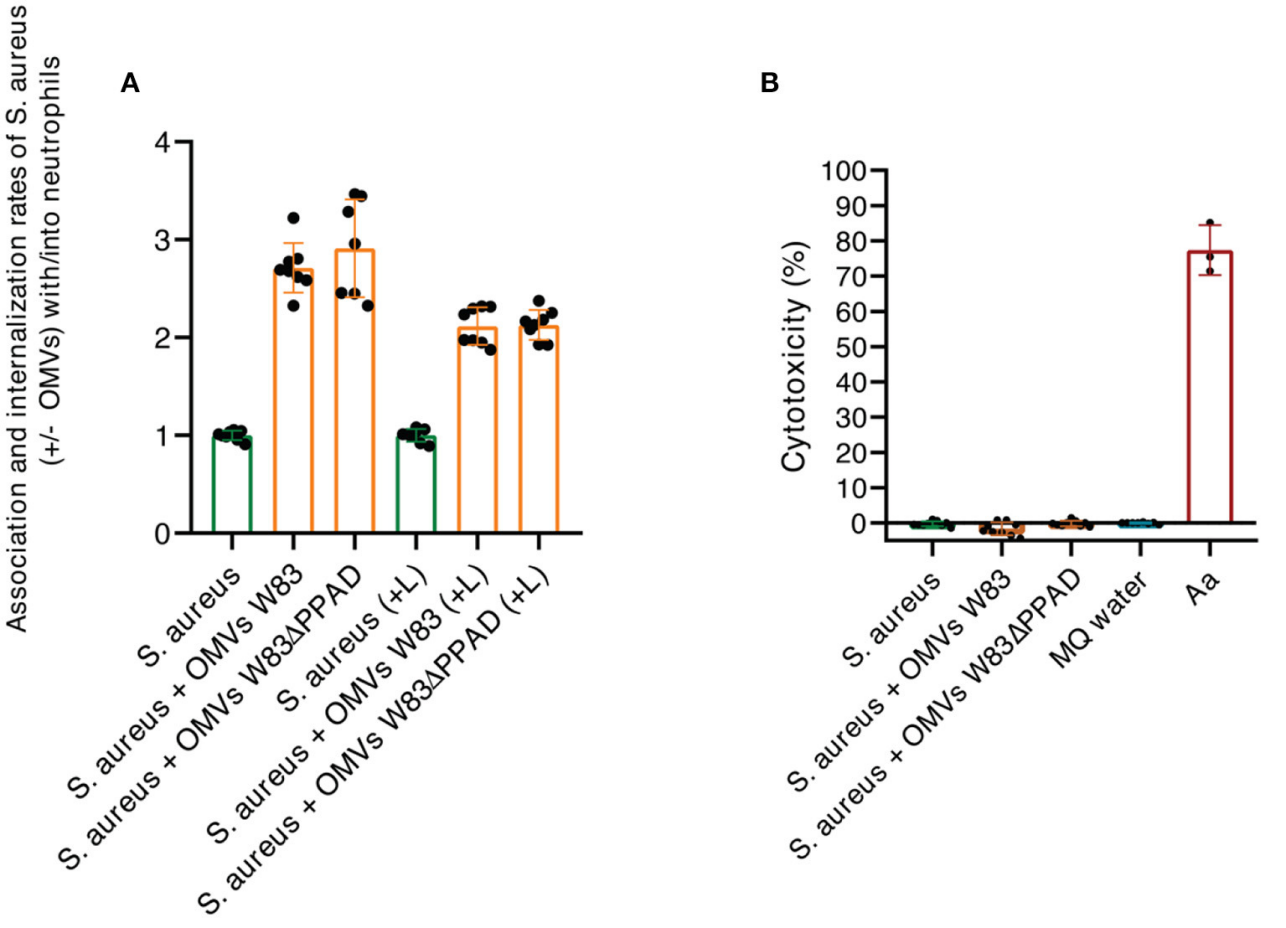
Characterization of neutrophil internalization of *S. aureus* upon exposure to *P. gingivalis* OMVs. **(A)** Ratios of the association/internalization indices of *S. aureus* incubated with OMVs and the association/internalization indices of *S. aureus* incubated without OMVs. L, lysostaphin. **(B)** Percentage of neutrophil killing as determined by LDH release. Neutrophils were challenged with *S. aureus* in the presence or absence of OMVs from *P. gingivalis* W83 or W83ΔPPAD. *A. actinomycetemcomitans* 30R **(Aa)** was used as a positive control for cytotoxicity and MilliQ water as a negative control for spontaneous LDH release from the neutrophils. The mean and standard deviations were calculated from three biological replicates and two technical replicates.

The known association between rheumatoid arthritis and SAB, coupled with our present findings, begged the question of whether *P. gingivalis* OMVs can influence the interaction of *S. aureus* with the human immune system. To address this question, we investigated the effects of *P. gingivalis* OMVs on the interactions of *S. aureus* with human neutrophils, which form a primary innate defense line against both *S. aureus* and *P. gingivalis* infections [1, 31]. In particular, we pre-incubated *S. aureus* HG001 with OMVs of either *P. gingivalis* W83 or W83ΔPPAD, exposed the OMV-treated bacteria to human neutrophils, and inspected the bacteria-neutrophil interactions by confocal microscopy. The results are presented in Figure 5, where the nuclei of neutrophils are stained with DAPI (blue) and the actin filaments with phalloidin-TRITC (yellow), whereas the *S. aureus* bacteria are tagged with GFP (green) and the *P. gingivalis* OMVs are labeled with specific antibodies (red). Aggregation of *S. aureus* due to the exposure to *P. gingivalis* OMVs of either strain W83 or W83ΔPPAD was clearly detectable, as was the internalization of *S. aureus* by the neutrophils (Figure 5A; Supplementary Figures S1,S2). Interestingly, analysis by flow cytometry showed that OMV exposure promoted the adhesion of *S. aureus* to the neutrophils, and the subsequent bacterial internalization by the neutrophils (Figure 6A). Notably, even though the OMVs of *P. gingivalis* effectively coated the *S. aureus* cells prior to internalization by the neutrophils, the OMVs did not appear to be internalized together with the *S. aureus* cells (Figures 5B,C). To verify that the neutrophil viability was not affected by the presence of OMVs, a cytotoxicity assay was performed that is based on the release of LDH by dying cells. The results of this assay showed that *S. aureus* HG001 had, neither in the absence nor in the presence of *P. gingivalis* OMVs, a negative impact on the viability of the neutrophils. In contrast, exposure of the neutrophils to the oral pathogen *A. actinomycetemcomitans* 30R, which has strong leukotoxic activity [27], resulted in a high degree of neutrophil killing and subsequent LDH release (Figure 6B). Altogether, these observations show that *P. gingivalis* OMVs promote the phagocytosis of *S. aureus* cells. This could represent an effective strategy of *P. gingivalis* to eliminate an unwanted competitor, or even an immune evasive strategy where enhanced phagocytosis of another bacterium would distract the neutrophil's attention from *P. gingivalis*.

## Conclusion

Since *S. aureus* has been identified in dental biofilms and saliva, previous studies have started to explore the effects of the *P. gingivalis* strain ATCC3277 and/or its OMVs on this opportunistic pathogen [17–21]. Our present observations using the highly virulent *P. gingivalis* strain W83 underscore the significant effects of *P. gingivalis* OMVs on *S. aureus*, showing for the first time that the OMV-induced aggregation of *S. aureus* is reversible and that it relies on the concerted action of gingipains and PPAD. In particular, the involvement of PPAD in *S. aureus* aggregation is a novel notion, as previous studies had merely hypothesized a role only for gingipains [17] based on the presence of hemagglutinin adhesion domains in these enzymes [34]. The presently reported synergy of PPAD and gingipains suggests that citrullination is involved in the OMV-mediated aggregation of *S. aureus*, as gingipain-catalyzed protein cleavage exposes carboxyl-terminal arginine residues that are the preferential targets of PPAD [11, 14]. However, to fully understand this phenomenon it will be necessary to dissect which components of the *P. gingivalis* OMVs interact with which components of *S. aureus* to bring about the staphylococcal aggregation. The second novel observation in our present studies is that the OMV-promoted aggregation of *S. aureus* stimulates the binding and internalization of this bacterium by neutrophils. This could clearly be beneficial from the perspective of *P. gingivalis*, since it may help in the elimination of an unwanted competitor. However, the delivery of *S. aureus* to neutrophils may not be without consequences for the human host, as there is ample evidence for the effective survival of *S. aureus* inside neutrophils and other immune cells, which may even serve as ‘Trojan horses' to carry *S. aureus* to other sites in the human body [1]. Importantly, the immune cell-facilitated trafficking of *S. aureus* includes the blood stream. Thus, the *P. gingivalis* OMV-mediated delivery of *S. aureus* to a professional phagocyte like the neutrophil might explain, at least in part, why the autoimmune disease RA is a risk factor for the deadly SAB [9, 10]. However, one has to bear in mind that the possible relationships between *P. gingivalis, S. aureus*, RA and SAB, are based on suppositions. Further research on the tripartite interactions between *P. gingivalis, S. aureus*, and human immune cells will, therefore, be needed to fully comprehend the associations between rheumatoid arthritis and SAB. For instance, this would include studies on the survival of *S. aureus* upon *P. gingivalis* OMV-mediated internalization by neutrophils. Such research may potentially open up novel avenues for the prevention or treatment of both diseases.

## Data availability statement

The raw data supporting the conclusions of this article will be made available by the authors, without undue reservation.

## Ethics statement

The studies involving human participants were reviewed and approved by Medical Ethical Committee of the University Medical Center Groningen (approval no. Metc2012-375). The patients/participants provided their written informed consent to participate in this study.

## Author contributions

MT, AH, YF, and ML-Á: sample collection, sample and data processing, and laboratory work. MT, AH, GG, and JD: study design and manuscript preparation. All authors contributed to the article and approved the submitted version.

## Conflict of interest

The authors declare that the research was conducted in the absence of any commercial or financial relationships that could be construed as a potential conflict of interest.

## Supplementary material

Supplementary Figure S1 *S. aureus* interaction with neutrophils in the presence of OMVs. Confocal fluorescence microscopy images of neutrophils infected with *S. aureus* HG001 in the absence or presence of OMVs from *P. gingivalis* W83 or W83ΔPPAD. DAPI was used to stain the nuclei of neutrophils and Phalloidin-TRITC to stain actin. *S. aureus* was visualized based on the expression of GFP from pJL-sar-GFP and OMVs were labeled with specific antibodies (scale bars = 50 μm). Each experiment was repeated at least twice.

Supplementary Figure S2 OMV-facilitated association and internalization of *S. aureus* in the presence of OMVs. **(A)** Confocal fluorescence microscopy images of neutrophils infected with *S. aureus* HG001 in the absence or presence of OMVs from *P. gingivalis* W83 or W83ΔPPAD. White arrows mark internalized *S. aureus* bacteria (scale bar = 30 μm). Each experiment was repeated at least twice. **(B)** *S. aureus* association and internalization indices of neutrophils infected with *S. aureus* in the presence of OMVs from *P. gingivalis* W83 or W83ΔPPAD. L, lysostaphin. The mean and standard deviations were calculated from three biological replicates and two technical replicates.
