# Supplementary figures and images for: Outer membrane vesicles of the oral pathogen Porphyromonas gingivalis promote aggregation and phagocytosis of Staphylococcus aureus
Source: Front Oral Health. 2022 Jul 22;3:948524. doi: 10.3389/froh.2022.948524 (PMC9354530; doi:10.3389/froh.2022.948524)

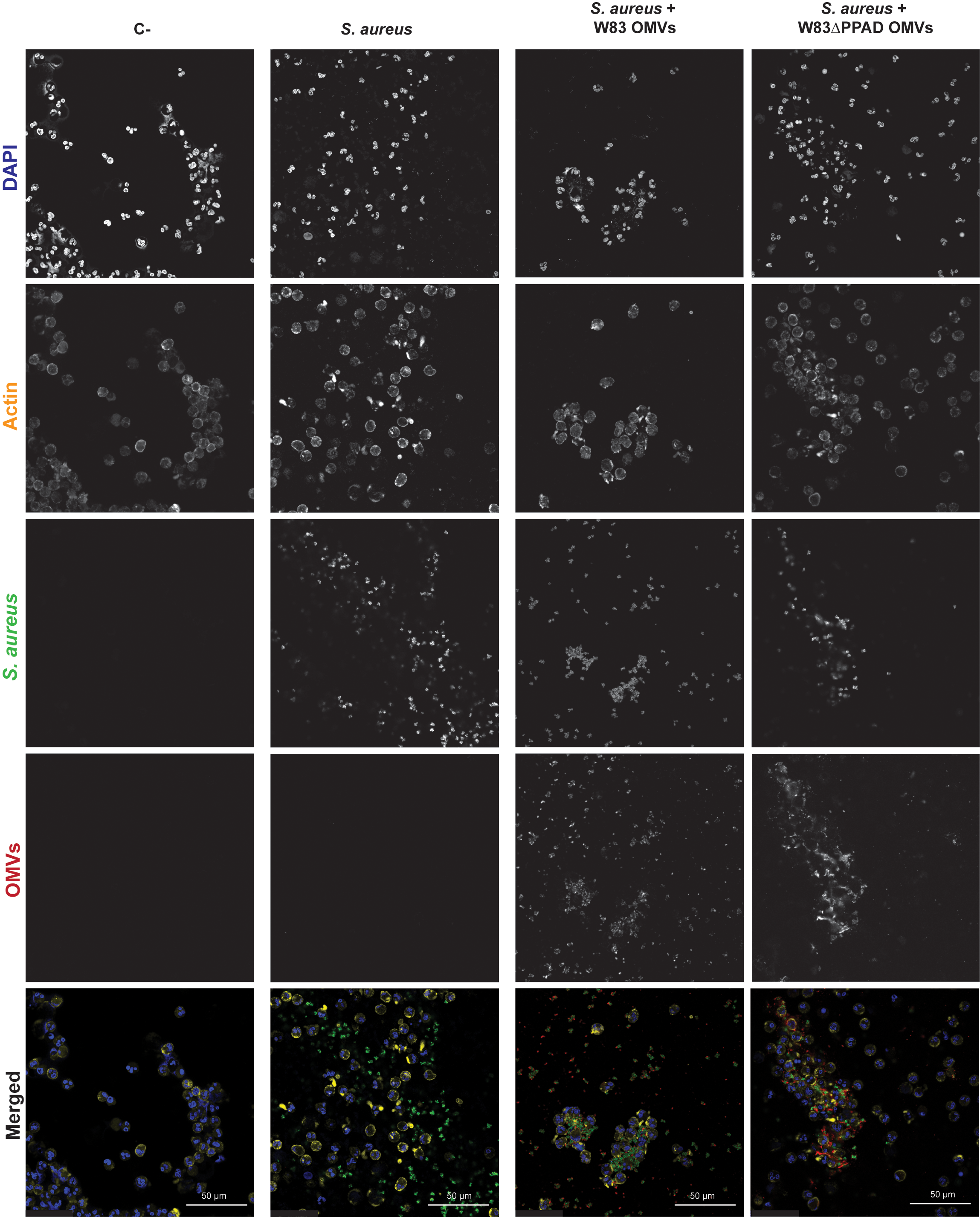

Supplement: Supplementary Figure S1 — S. aureus interaction with neutrophils in the presence of OMVs. Confocal fluorescence microscopy images of neutrophils infected with S. aureus HG001 in the absence or presence of OMVs from P. gingivalis W83 or W83ΔPPAD. DAPI was used to stain the nuclei of neutrophils and Phalloidin-TRITC to stain actin. S. aureus was visualized based on the expression of GFP from pJL-sar-GFP and OMVs were labeled with specific antibodies (scale bars = 50 μm). Each experiment was repeated at least twice. [file Image_1.TIF]

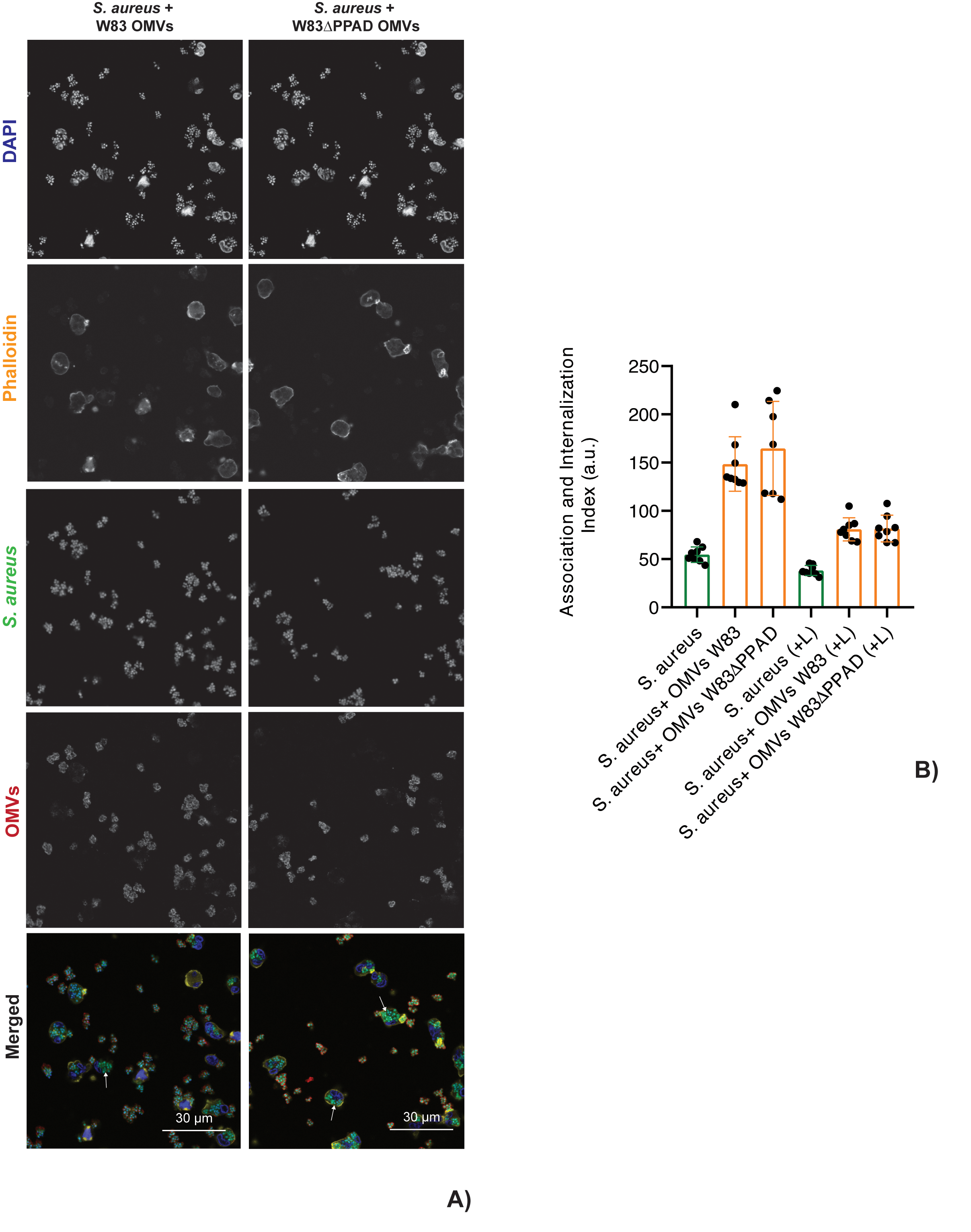

Supplement: Supplementary Figure S2 — OMV-facilitated association and internalization of S. aureus in the presence of OMVs. (A) Confocal fluorescence microscopy images of neutrophils infected with S. aureus HG001 in the absence or presence of OMVs from P. gingivalis W83 or W83ΔPPAD. White arrows mark internalized S. aureus bacteria (scale bar = 30 μm). Each experiment was repeated at least twice. (B) S. aureus association and internalization indices of neutrophils infected with S. aureus in the presence of OMVs from P. gingivalis W83 or W83ΔPPAD. L, lysostaphin. The mean and standard deviations were calculated from three biological replicates and two technical replicates. [file Image_2.TIF]
